# Whole-genome sequence analysis of clinically isolated carbapenem resistant *Escherichia coli* from Iran

**DOI:** 10.1186/s12866-023-02796-y

**Published:** 2023-02-27

**Authors:** Mehri Haeili, Samaneh Barmudeh, Maryam Omrani, Narges Zeinalzadeh, Hossein Samadi Kafil, Virginia Batignani, Arash Ghodousi, Daniela Maria Cirillo

**Affiliations:** 1 Department of Animal Biology, Faculty of Natural Sciences, University of Tabriz, Tabriz, Iran; 2 IRCCS San Raffaele Scientific Institute, Milan, Italy; 3 Drug Applied Research Center, Faculty of Medicine, Tabriz University of Medical Sciences, Tabriz, Iran; 4 Emerging Bacterial Pathogens Unit, Division of Immunology, Transplantation and Infectious Diseases, IRCCS San Raffaele Scientific Institute, Milan, Italy; 5 Vita-Salute San Raffaele University, Milan, Italy

**Keywords:** Carbapenem resistance, *Escherichia coli*, NDM-1, NDM-5, Whole genome sequencing, Epidemiology

## Abstract

**Background:**

The emergence of carbapenem-resistant *Enterobacterales* (CRE) continues to threaten public health due to limited therapeutic options. In the current study the incidence of carbapenem resistance among the 104 clinical isolates of *Escherichia coli* and the genomic features of carbapenem resistant isolates were investigated.

**Methods:**

The susceptibility to imipenem, tigecycline and colistin was tested by broth dilution method. Susceptibility to other classes of antimicrobials was examined by disk diffusion test. The presence of *bla*_OXA-48_, *bla*_KPC_, *bla*_NDM_, and *bla*_VIM_ carbapenemase genes was examined by PCR. Molecular characteristics of carbapenem resistant isolates were further investigated by whole-genome sequencing (WGS) using Illumina and Nanopore platforms.

**Results:**

Four isolates (3.8%) revealed imipenem MIC of ≥32 mg/L and positive results for modified carbapenem inactivation method and categorized as carbapenem resistant *E. coli* (CREC). Colistin, nitrofurantoin, fosfomycin, and tigecycline were the most active agents against all isolates (total susceptibility rate of 99, 99, 96 and 95.2% respectively) with the last three compounds being found as the most active antimicrobials for carbapenem resistant isolates (susceptibility rate of 100%). According to Multilocus Sequence Type (MLST) analysis the 4 CREC isolates belonged to ST167 (*n =* 2), ST361 (*n =* 1) and ST648 (*n =* 1). NDM was detected in all CREC isolates (NDM-1 (*n =* 1) and NMD-5 (*n =* 3)) among which one isolate co-harbored NDM-5 and OXA-181 carbapenemases. WGS further detected *bla*_CTX-M-15_, *bla*_CMY-145_, *bla*_CMY-42_ and *bla*_TEM-1_ (with different frequencies) among CREC isolates. Co-occurrence of NDM-type carbapenemase and 16S rRNA methyltransferase RmtB and RmtC was found in two isolates belonging to ST167 and ST648. A colistin-carbapenem resistant isolate which was *mcr*-negative, revealed various amino acid substitutions in PmrB, PmrD and PhoPQ proteins.

**Conclusion:**

About 1.9% of *E. coli* isolates studied here were resistant to imipenem, colistin and/or amikacin which raises the concern about the outbreaks of difficult-to-treat infection by these emerging superbugs in the future.

**Supplementary Information:**

The online version contains supplementary material available at 10.1186/s12866-023-02796-y.

## Background

Carbapenems are often one of the last resort options available to clinicians for treatment of serious infections caused by multidrug-resistant (MDR) Gram-negative bacteria including *Enterobacterales* [1]. However, the clinical overuse of carbapenem agents inevitably resulted in increased drug resistance and emergence of carbapenem resistant *Enterobacterales* (CRE). Currently, the CRE infections have become an important focus of infection control due to limited therapeutic choices and deleterious outcomes for patients [2, 3]. Treating these infections are considered challenging and often requires the use of older agents, such as tigecycline or colistin, which is frequently associated with unclear efficacy and/or toxicity issues [4, 5]. Carbapenem non-susceptibility in CRE is mainly related to enzymatic hydrolysis of antibiotics by carbapenemase (s), and to lesser extent production of dysfunctional entry routes of carbapenems (e.g. mutated porins) or overexpressed efflux pumps [5]. Clinically relevant carbapenemases belong to Class A *Klebsiella pneumoniae* carbapenemase [KPC]; Class B metallo-β-lactamases (MBLs) such as New Delhi MBL (NDM), and imipenemase (IMP); Verona integron-encoded MBL (VIM), and Class D oxacillinases [OXA enzymes] such as OXA-48-like carbapenemases. Class A and D β-lactamases each utilize serine at the active site whereas Class B enzymes require divalent cations usually Zn^2+^ ion as metal cofactor to catalyze the hydrolysis of β-lactams [5, 6]. The MBLs are a source of great concern as they hydrolyze all β-lactams except monobactams including aztreonam, and are not inhibited by the commercially available β-lactamase inhibitors such as avibactam, clavulanate, sulbactam, and tazobactam [7]. Moreover, MBLs are often coproduced with other β-lactamases that can hydrolyze aztreonam or even with other determinants conferring resistance to aminoglycosides and fluoroquinolones resulting in multidrug resistance phenotype [7, 8].

The geographic distribution of MBL enzymes among CRE is substantially diverse. According to a study on isolates from 40 countries conducted between 2012 and 2014, 44.2% of MBL-positive isolates of *Enterobacteriaceae* were found to carry *bla*_NDM_, 39.3% carried *bla*_VIM_, and 16.5% harbored *bla*_IMP_ [7]. *Escherichia coli*, as one of the most problematic species of *Enterobacterales* is an opportunistic pathogen and a common cause of urinary tract, enteric and bloodstream infections worldwide [9]. There is limited information regarding the incidence and the type of carbapenemases mediating resistance to these last-resort agents among *E. coli* isolates from Iran [10, 11]. Proper monitoring of the drug resistance patterns in the circulating bacteria and unraveling mechanisms conferring carbapenem resistance would provide valuable information for designing appropriate antibiotic prescription guidelines and subsequently better infection control strategies. Therefore, we aimed to investigate the drug susceptibility pattern of *E. coli* isolates obtained from different clinical samples, to identify prevalence and genotypes of carbapenem resistant isolates.

## Results

### Bacterial isolates and antimicrobial susceptibility testing

In the current work we studied 104 *E. coli* isolates obtained from a large referral hospital in an effort to determine the incidence of carbapenem resistance and the genomic features of resistant clones. According to imipenem susceptibility testing results performed by broth macrodiltion, and interpreted by CLSI guidelines, 94% of isolates (*n =* 98) were characterized with imipenem MICs ≤1 mg/L and were categorized as susceptible. While two (1.9%) and four (3.8%) isolates with imipenem MICs of 2 and ≥ 32 mg/L were categorized as intermediate and resistant respectively (Table 1). A total of 99% (*n =* 103) and 95.2% (*n =* 99) of isolates had colistin and tigecycline MICs of ≤2 and ≤ 0.5 mg/L, respectively and were categorized as susceptible to the these antimicrobials (Table 1). The susceptibility rate to other antimicrobials tested by disk diffusion are presented in Table 2. The four imipenem resistant isolates which also showed resistance to meropenem, revealed positive results for Modified Carbapenem Inactivation Method (mCIM) ((supplementary Fig. 1) and were designated as carbapenem resistant *E. coli* (CREC). Moreover, all imipenem susceptible (*n =* 98) and intermediate isolates (*n =* 2) were found to be also susceptible to meropenem. In addition, the two imipenem intermediate-meropenem susceptible isolates as well as 20 randomly selected imipenem-meropenem susceptible isolates showed negative results for mCIM. Since *Enterobacterales* with imipenem MICs ≤2 mg/L are categorized as susceptible according to EUCAST breakpoints, the above mentioned two isolates (with imipenem MIC = 2 mg/L) were also categorized as carbapenem susceptible *E. coli* (CSEC) along with other imipenem-meropenem susceptible isolates. Overall, colistin, nitrofurantoin, fosfomycin and tigecycline were the most active agents against all studied isolates (total susceptibility rate of 99, 99, 96, and 95.2% respectively) with the last three compounds being found as the most effective antimicrobials for carbapenem resistant isolates (susceptibility rate of 100%). Among the CSEC, 10% were characterized with no resistance to tested antibiotics and 11, 15, 23, 21 and 20% showed resistance to one, two, three, four and five or more antibiotics respectively. On the other hand, all CREC isolates were found to be resistant to at least three different tested antimicrobial agents (in addition to β-lactams).

**Table 1:** The MIC values of the antibiotics tested for clinical isolates of *Escherichia coli*

Antibiotic	Number of isolates with MICs (mg/L)					
	≤0.12	0.25	0.5	1	2	≥4
Imipenem	8	52	31	7	2	4
Colistin	0	29	38	28	8	1
Tigecycline	10	36	53	5	0	0

**Table 2:** Antimicrobial susceptibility pattern of clinical isolates of *E. coli* determined by disk diffusion

Antimicrobial agent	ISEC (n/%)			IREC (n(%))			Total (n(%))		
	S	I	R	S	I	R	S	I	R
Meropenem	100	0	0	0	0	4 (100)	100 (96)	0	4 (3.8)
Fosfomycin	96	0	4	4 (100)	0	0	100 (96)	0	4 (3.8)
Nitrofurnatoin	99	0	1	4 (100)	0	0	103 (99)	0	1 (0.96)
Amikacin	93	2	5	2 (50)	0	2 (50)	95 (91.3)	2 (1.92)	7 (6.7)
Gentamicin	70	0	30	1 (25)	0	3 (75)	71 (68.2)	0	33 (31.7)
Tetracycline	32	0	68	2 (50)	0	2 (50)	34 (32.6)	0	70 (67.3)
Doxycycline	30	7	63	2 (50)	0	2 (50)	32 (30.7)	7 (6.7)	65 (62.5)
Chloramphenicol	80	1	19	2 (50)	0	2 (50)	82 (78.8)	1 (0.96)	21 (20.1)
Ciprofloxacin	26	4	70	0	0	4 (100)	26 (25)	4 (3.8)	74 (71.1)
Ceftazidime	50	20	30	0	0	4 (100)	50 (48)	20 (19.2)	34 (32.6)

*ISEC* Imipenem susceptible *E. coli* (also includes isolates with intermediate susceptibility to imipenem (MIC = 2 mg/L)), *IREC* Imipenem Resistant *E. coli*, *S* Susceptible, *I* Intermediate, *R* Resistant

### Genotypic antimicrobial resistance

PCR screening of 4 carbapenemase genes was performed for all isolates (*n =* 6) with imipenem MIC ≥2 mg/l. The *bla*_NDM_ (*n =* 4 isolates) and *bla*_OXA-48-like_ (*n =* 1 isolate) were the only carbapenemase encoding genes detected among CREC isolates and the absence of other carbapenemases in the studied isolates was confirmed by WGS analysis. In two isolates with imipenem MICs of 2 mg/L none of the studied carbapenemase genes were detected by PCR. According to WGS analysis, *bla*_NDM_ (NDM-1 and NDM-5) was detected in all CREC isolates with *bla*_NDM-5_ variant being found in three isolates. The OXA-181 variant was detected in one isolate co-harboring NDM-5 enzyme. Multilocus Sequence Type (MLST) analysis showed that the four CREC isolates belonged to ST167 (*n =* 2), ST361 (*n =* 1) and ST648 (*n =* 1) (Table 3). The CREC isolates harbored one or more β-lactamases such as CTX-M-15, CMY-145, CMY-42 and TEM-1*.* Two CREC isolates belonging to ST167 and ST648 harbored 16S rRNA methytrnsferaseand (RMTase) RmtB and RmtC conferring resistance to all clinically relevant aminoglycosides. The genes associated with resistance to other antimicrobials including tetracyclines, sulfonamide, aminoglycosides and quinolones are presented in Table 4. Analysis of chromosomally-encoded proteins involved in antimicrobial resistance revealed amino acid substitutions in quinolone resistance-determining regions (QRDR) in GyrA (S83L, D87N) and ParC (S80I) which was consistent with quinolone resistance phenotype in all CREC isolates. In a carbapenem-colistin resistant isolate (colistin MICs of 4 mg/L), no plasmid born colistin resistance *mcr*-type gene was detected. However, whole genome sequencing revealed various chromosomal point mutations in PmrB (H2R, G19R, D283G, and A360V), PhoQ (L467M), PhoP (I44L) and PmrD (N11D, M20K, A27T, K82Q, V83S) proteins.

**Table 3:** Genomic and phenotypic characteristics of carbapenem resistant *E. coli* isolates

Isolate	Clinical sample	Broth dilution MIC (mg/L)			Sequence type	Carbapenemase	ASP^**a**^	
							Susceptible	Resistant
		IMP	COL	TGC				
CREC-17	urine	32	4	0.12	648	NDM-5, OXA-181	FOS, NIT, TET, DOX, CHL, TGC	GEN, AMK, CIP, COL, IMP
CREC-18	urine	64	0.25	0.25	167	NDM-5	FOS, NIT, GEN, AMK, COL, TGC	CIP, TET, DOX, CHL, IMP
CREC-19	urine	64	0.25	0.25	167	NDM-1	FOS, NIT, CHL, COL, TGC	GEN, AMK, CIP, TET, DOX, IMP
CREC-20	blood	64	0.25	0.25	361	NDM-5	FOS, NIT, AMK, TET, DOX, COL, TGC	GEN, CIP, CHL, IMP

^a^Antimicrobial susceptibility profile, determined by disc diffusion and broth dilution methods

*IMP* Imipenem, *TGC* Tigecycline, *COL* Colistin, *FOS* Fosfomycin, *NIT* Nitrofurantoin, *GEN* Gentamicin, *AMK* Amikacin, *CHL* Chloramphenicol, *TET* Tetracycline, *DOX* Doxycycline, *CIP* Ciprofloxacin

**Table 4:** Antimicrobial resistance and virulence genes identified by WGS among the carbapenem resistant *E. coli* isolates

Isolate	Antimicrobial Resistance Genes								Virulence Genes
	Aminoglycoside	Beta-lactam	Chloramphenicol	Macrolide	Quinolone	Sulfonamide	Trimethoprim	Tetracycline	
**CREC-17**	*rmtB, aadA2*	***bla***_**OXA-181**_***, bla***_**NDM-5,**_ *bla*_CMY-42_*, bla*_TEM-1_*,*	–	*erm(B),mph(A)*	*qnrS1, gyrA* (S83L, D87N)*, parC* (S80I), *parE* (S458A)	*sul1*	*dfrA12*	–	*fyuA, irp2, air, eilA, kpsE, yfcV, lpfA, gad, chuA, traT, terC*
**CREC-18**	*aadA5, aph(3″)-Ib, aph(6)-Id*	***bla***_**NDM-5,**_ *bla*_OXA-1_*, bla*_CTX-M-15_*,*	*catA1, catB3*	*mph(A)*	*aac*(*6′*)*-Ib-cr* *gyrA* (S83L, D87N)*, parC* (S80I), *parE* (S458A)	*sul1*	*dfrA17*	*tetA*	*fyuA, irp2, hylE, csgA, yehA, yehB, yehC, yehD, hha gad, iss, fdeC, traT terC*
**CREC-19**	*aac(6′)-Ib3, rmtC, aadA2*	***bla***_**NDM-1,**_ *bla*_CMY-145_*, bla*_CTX-M-15_*,*	–	*erm(B), mph(A)*	*qnrS1, gyrA* (S83L, D87N)*, parC* (S80I), *parE* (S458A)	*sul1*	*dfrA12*	*tetA*	*iucC, iutA, hlyE, csgA, yehB, yehC, yehD,hha, capU, gad, iss, fdeC, hra, terC*
**CREC-20**	*aac(3)-IId, aph(3′)-Ia, aadA1,aadA2*	***bla***_**NDM-5,**_ *bla*_CTX-M-15_	*cmlA1*	*mph(A)*	*gyrA* (S83L, D87N)*, parC* (S80I, E84G)	*sul1, sul3*	*dfrA12*	–	*hylE, csgA, shiA, sitA, yehA, yehB, yehC, colE8, colE2, fimH, capU, gad, fdeC, hra, traT, traJ, terC*

Genomic analysis of virulence genes, revealed presence of genes belonging to several functional categories including those involved in iron acquisition (*irp2*, *fyuA*, *chuA*, *iucC*, *iutA*, *sitA*), adherence (*lpfA*, *yfcV*, *csgA*, *fdeC, yeh* fimbrial genes, *air, fimH*), serum survival (*iss*, *traT*), tellurite resistance (*terC),* production of toxin (*hlyE*), capsular polysaccharide *(kpsE)*, and colicin (*colE2*, *colE8*) (Table 4).

### Whole-genome assembly and genome visualization of CREC strains

Illumina short read and Oxford Nanopore long read data were used as input of hybrid de novo assembly to reconstruct the complete CREC genomes. The Unicycler-short reads/ Ratatosk corrected long reads hybrid assembly resulted in closed chromosomes for all three isolates. The genomic characteristics of all three assembled genomes are shown in supplementary Table 1.

Moreover, to understand the relevance of the assembled genomes of this study in comparison to global genomes, a comparative genome analysis was performed with other complete *E. coli* genomes of ST648 (GenBank Accessions CP048107, as reference genome), ST167 (GenBank Accessions CANDYB000000000) and ST361 (GenBank Accessions CP103704) (supplementary Fig. 2 & supplementary Table 2).

### Gene composition analysis of the plasmids in CREC strains

**Fig. 1:**
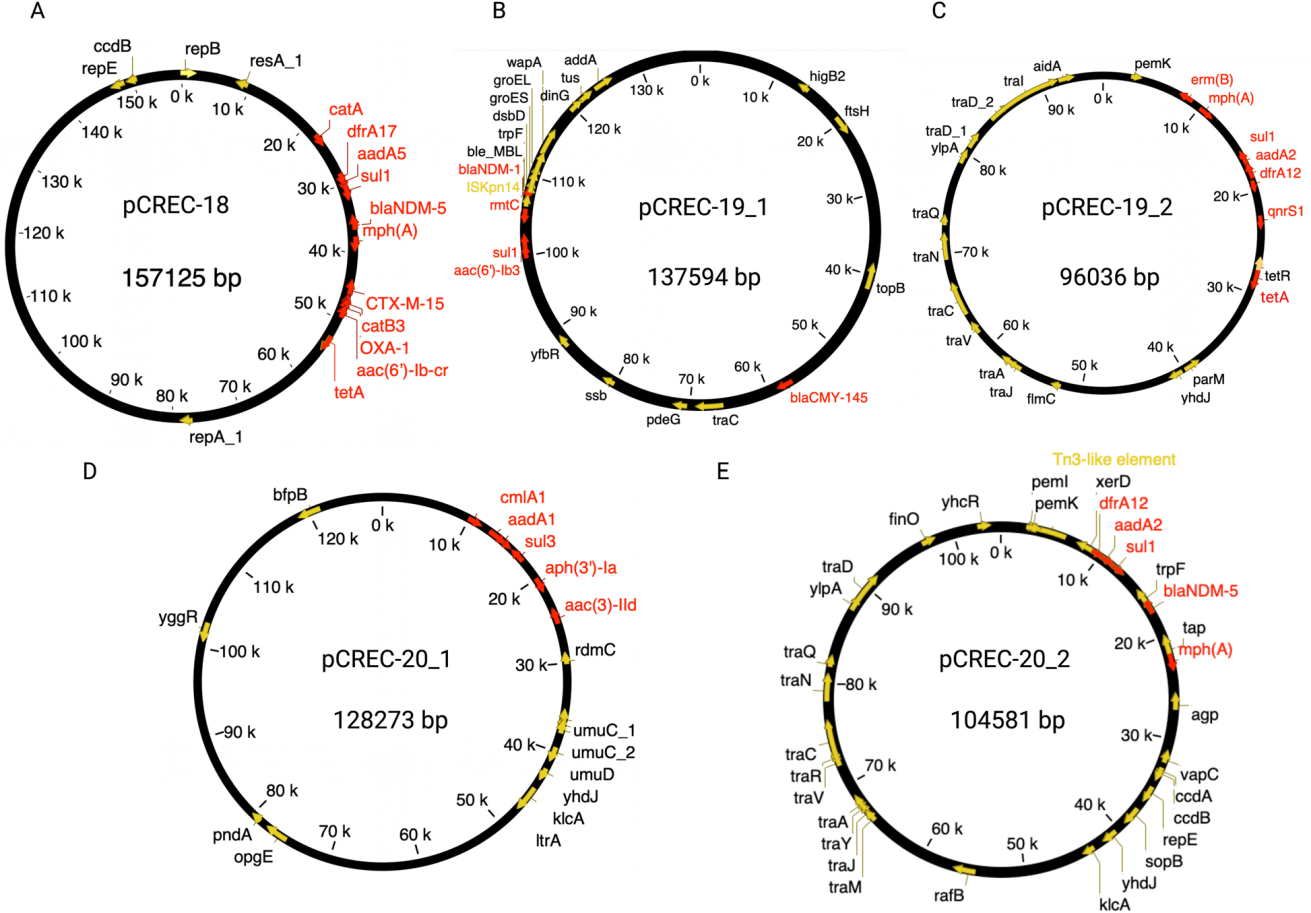
Circular representation of the studied plasmids. The arrows around the map indicate deduced ORFs and their orientation. Certain important genes are also indicated on the ring. The schematics were generated through the ‘A plasmid Editor‘(APE) program

In this study, six closed complete plasmids were detected in three CREC strains and were further analyzed. The CREC-18 strain had an IncF-like plasmid, pCREC-18 (157,125 bp) which co-harbors the *bla*_NDM-5_ and genes conferring resistance to other antimicrobials as presented in Fig. 1A. BLASTn of the pCREC-18 sequence showed high similarity to other *bla*_NDM-5_ carrying plasmids from clinical isolates i.e. NDM-5- IncFIA plasmid in a clinical *E. coli* isolate (CP083875.1).

The strain CREC-19 harbored two different plasmids, an IncA/C2-like plasmid (pCREC-19_1, 137,594 bp) co-harboring *bla*_NDM-1_, *rmtC* and additional resistance conferring genes (Fig. 1B) where *bla*_NDM-1_ was located in a mobile region with a structure of rmtc-ISKpn14-blaNDM1-bleMBL-trpF-tat-dsbC-groES-groEL as reported in other studies [12, 13]. BLASTn of this plasmid showed high similarity to other *bla*_NDM-1_ carrying plasmids found in other *Enterobacterales* i.e. NDM-1-IncA/C2 plasmid in a clinical *Klebsiella pneumoniae* isolate (CP050164.1).

The second plasmid in CREC-19 was an IncFII-like plasmid (pCREC-19_2, 96,036 bp) carrying *qnrS1, sul1, tetA and* other antibiotic resistance genes (ARGs) as indicated in Fig. 1C.

Strain CREC-20 carried three plasmids: The plasmid pCREC-20_1 (128,273 bp) harboring *cmlA1*, *sul3*, *aadA1* and other ARGs was a type of IncB/O/K/Z plasmid (Fig. 1D). In addition, and similar to CREC-18, this strain also contains a *bla*_NDM-5_ containing IncF-like plasmid (104,581 bp) that carries other ARGs including *sul1, dfrA12* and *aadA2* (Fig. 1E) but, the overall structure of the two NDM-5-producing plasmids were different (Supplementary Fig. 3). Finally, the CREC-17 also contained plasmid(s) carrying *bla*_NDM-5_ and other resistance conferring genes. However, the long read sequencing was not successful and no closed plasmid(s) were achieved for this sample relying only on the short reads as input for assembly. The genome information of all detected plasmids are summarized in Table 5.

**Table 5:** Features of plasmids identified in CREC-18, CREC-19 and CREC-20 isolates

Plasmid	Plasmid replicon type	pMLST sequence type	Size (bp)	GC content (%)	Accession numbers	CDS	Antimicrobial resistance genes ^**a**^
pCREC-18	IncFIA	F31:A1:B58	157,125	52.3	CP107063	169	*bla*_NDM-5_, *bla*_CTX-M-15,_, *bla*_OXA-1_, *sul1, mph(A)*, *aac(6′)-Ib-cr, aadA5, dfrA17, catA1, catB3, tetA*
pCREC-19_1	IncA/C2	1	137,594	52	CP107062	171	*bla*_NDM-1_, *rmtC*, *sul1*, *aac(6′)-Ib3, bla*_CMY-145_
pCREC-19_2	IncFII	F2:A-:B-	96,036	52.9	CP107061	111	*erm(B)*, *mph(A*), *sul1*, *aadA2*, *dfrA12*, *qnrS1, tetA*
pCREC-20_1	IncB/O/K/Z	Unknown	128,273	51.6	CP107060	147	*cmlA1*, *aaA1*, *sul3*, *aph(3′)-Ia, aac(3)-IId*
pCREC-20_2	IncFIA	F2:A4:B-	104,581	52.4	CP107059	112	*aadA2*, *sul1*, *bla*_NDM-5_, *mph(A), dfrA12*
pCREC-20_3	IncY	Unknown	94,240	47.9	CP107058	107	–

^a^*aac* Aminoglycoside acetyltransferase, *aad* Aminoglycoside adenylyltransferase, *dfrA* Dihydrofolate reductase, *aph *Aminoglycoside phosphotransferase, *erm* Erythromycin ribosome methylase, *tetA* Tetracycline resistance, *mph* Macrolide phosphotransferases, *rmtC* Ribosomal RNA methyltransferase, *sul* Sulfonamide resistance gene, *cmlA* Chloramphenicol efflux transporter, *catA* Chloramphenicol acetyl transferase, *qnrS* Quinolone resistance

## Discussion

MDR Gram-negative bacteria are a major global public health threat, with an increasing incidence of CRE infections, for which therapeutic choices are limited. The most common mechanism of carbapenem resistance in CRE is production of carbapenemase enzymes which are encoded by genes located on plasmids, making them readily transferable [14]. In the current study the rate of carbapenem resistance among the studied isolates was found to be 3.84% (*n =* 4). MLST showed that the two CREC isolates (CREC-18 and 19) belonged to ST167, and the remaining 2 isolates were characterized with ST361 (CREC-20) and ST648 (CREC-17). The *E. coli* ST167 clone is emerging throughout the world, being frequently recognized for having a link with carbapenem resistance [15, 16] and for being involved in the transmission of the *bla*_NDM-1_ and *bla*_NDM-5_ genes in hospitals [17]. It has been demonstrated that the ST167 has peculiar virulence characteristics which facilitate its evolution to a high-risk clone with widespread colonization and infection capabilities [18]. Moreover, ST361 clone has been recently reported as the most frequent lineage of NDM-5 producing *E. coli* from Korea [19].

NDM was found in all CREC isolates with the NDM-5 being the most prevalent variant (*n =* 3). This is consistent with previous reports identifying the highest prevalence of NDM-positive *Enterobacterales* in the Indian subcontinent and the Middle East region [20]. Overall the prevalence of carbapenem resistant *E. coli* isolates in Iran is relatively low and a handful of studies have reported NDM-1 (the most prevalent), NDM-7 or OXA-181 producing *E. coli* isolates from the country [10, 11]. Our study would be the first report of clinical isolates of carbapenem-resistant *E. coli* carrying NDM-5 from Iran. Recently we reported NDM-5 producing *K. pneumoniae* isolates of clinical origin for the first time from Iran [21]. While NDM-1 is the most prevalent variant with widespread distribution among several members of *Enterobacterales* and other Gram-negative bacilli, NDM-5 appears to be more confined to *E. coli* isolates [22]. It has been described that NDM-producing strains are frequently resistant to a wide range of antimicrobial agents, due to co-harboring additional resistance determinants [22]. According to antimicrobial susceptibility testing results tigecycline, nitrofurantoin and fosfomycin were found as the most active agents against the CREC isolates. Colistin and tigecycline have been recognized as promising alternatives for treatment of CRE infections [23]. However, one CREC isolate (CREC17) in this study was characterized with colistin MIC of 4 mg/l and categorized as colistin resistant. Colistin resistance in *Enterobacterales* is found to be mainly mediated by acquisition of a plasmid-borne mobile colistin resistance (*mcr*) gene (encoding phosphoethanolamine transferase,) and/or chromosomal mutations within PhoPQ, PmrAB two component system or their regulatory gene *mgrB*, all of which result in LPS modifications [24]. The colistin-carbapenem resistant isolate identified in this study lacked an *mcr*-type gene but revealed chromosomal mutations within *pmrB*, *phoP*, *phoQ* and *pmrD* genes. Some of the detected amino acid variations have previously been reported from colistin resistant *E. coli* isolates including PmrB D283G [25], PmrB G19R [26], PmrB H2R/ A360V, PhoP I44L, PhoQ L467M and PmrD N11D/M20K/A27T /K82Q/V83S [27]. More importantly we found that two CRE isolates belonging to ST167 and ST648 co-harbored 16S rRNA methyltransferase RmtB/RmtC and NDM-type carbapenemases. The RMTases have been found to confer high-level and broad spectrum resistance to all clinically-relevant aminoglycosides (MIC > 256 mg/L) and to date ten 16S RMTases have been identified (namely ArmA, RmtA-H and NpmA)) [28]. Co-occurrence of RMTases and NDM-type carbapenemase has recently been reported in *Enterobacterales* from Europe [29, 30] and Latin America [31]. The emergences and spread of RMTase producing *Enterobacterales* is of great concern as the genes encoding RMTases are frequently located on plasmids along with those coding for ESBLs or carbapenemases rendering these bacteria resistant to multiple classes of antimicrobials used to treat MDR Gram-negative infections [30].

## Conclusion

Our study reports emergence of carbapenem resistant *E. coli* isolates harboring NDM-1, NDM-5 and OXA-181 variants. Although the prevalence of the CREC isolates were relatively low (< 4%) about 1.9% of *E. coli* isolates studied here were resistant to imipenem, quinolones, and colistin and/or amikacin which raises concerns about the future outbreak by highly resistant *Enterobacterales*. Considering the importance of aminoglycosides and polymyxins in the treatment of MDR *Enterobacterales*, resistance to these agents is considered as another emerging issue in addition to carbapenem resistance. Therefore, it is necessary to implement appropriate surveillance studies and infection control measures, to carefully monitor the prevalence of these pan- β-lactam-aminoglycoside-polymyxin resistant isolates in order to prevent the occurrence of outbreaks caused by these emerging superbugs.

## Materials and methods

### Study design and bacterial isolates

A total of 104 isolates of *E. coli* obtained from patients hospitalized at Imam Reza hospital, an 800-bed training hospital in Tabriz city (in period of June to September 2021) were studied. The clinical samples from which bacterial strains were isolated included urine, wound, blood and tracheal aspirates. Bacterial isolates were identified to species level using conventional biochemical methods including IMViC tests (indole test, methyl red test, Voges-Proskauer reaction, citrate utilization test), urease test, motility, ONPG (O-nitrophenyl-beta-D-galactopyranoside), reactions observed on Triple Sugar Iron (TSI) agar (H2S and gas production, carbohydrate utilization pattern) [32].

### Antimicrobial susceptibility testing

Susceptibility of the bacterial isolates to imipenem, colistin and tigecycline was performed by reference broth macrodilution methodology using imipenem, tigecycline hydrae and colistin sulphate powders from Glentham Life Sciences (Corsham, United Kingdom) and freshly prepared (less than 12-h-old) Mueller-Hinton broth from Difco (BD Diagnostic Systems, Sparks, MD, United States). Testing susceptibility to other antibiotics was performed by disc diffusion method (Kirby– Bauer) using the following antibiotics: meropenem, gentamicin, amikacin, ceftazidime, ciprofloxacin, tetracycline, doxycycline, chloramphenicol, nitrofurantoin and fosfomycin (BBL Sensi-DiscTM, Becton-Dickinson, Sparks, MD, United States). Clinical and Laboratory Standards Institute (CLSI) guidelines was used for data interpretation of all tested antimicrobials except for tigecycline and colistin. The European committee on antimicrobial susceptibility testing (EUCAST) criteria issued for *Enterobacterales* were applied for interpretation of tigecycline and colistin susceptibility results (tigecycline MIC > 0.5 mg/l, resistant; colistin MIC > 2 mg/l, resistant). *Escherichia coli* ATCC 25922 was used as a quality-control strain for antimicrobial susceptibility testing.

### Phenotypic detection of carbapenemase production by mCIM

The mCIM test was performed for all isolates with imipenem MIC≥2 mg/L or meropenem resistant isolates and 20 imipenem-meropenem susceptible isolates, which were selected randomly as negative controls. For each isolate to be tested a 1-μL loopful of bacteria from an overnight blood agar plate was resuspended in 2-mL tube of tryptic soy broth (TSB). A 10-μg meropenem disk was then placed in the suspension, and incubated at 35 °C for 4 h ± 15 min. Subsequently, the disks were removed and placed on MHA plates freshly inoculated with a 0.5 McFarland suspension of *E. coli* ATCC 25922 strain. The plates were incubated at 35 °C for 18 to 24 h. An inhibition zone diameter of 6–15 mm or colonies within a 16–18 mm zone was considered to be a positive result, and a zone of inhibition ≥19 mm was considered to be a negative result [33].

### Detection of carbapenemase encoding genes

The genomic DNA was extracted from the bacterial strains by boiling the lysates prepared from the strains as described previously [34]. The presence of four major carbapenemase-encoding genes, *bla*_KPC_, *bla*_VIM_, *bla*_NDM_, and *bla*_OXA-48-like_, was examined by PCR using the primers and amplification conditions described previously [35]. Clinical isolates of *Klebsiella pneumoniae* harboring 4 different carbapenemases (obtained from our microbial collection and confirmed for having carbapenemase gene by PCR and sequencing) were used as positive controls.

### Whole genome sequencing

All four CREC strains were shipped to the Emerging Bacterial Pathogens Unit, Division of Immunology, Transplantation and Infectious Diseases, IRCCS San Raffaele Scientific Institute, Milan, Italy in order to preform illumina short-read and Nanopore long-read whole genome sequencing.

### Illumina short-read DNA sequencing and analysis

In brief, genomic DNA from the CREC strains were extracted from MHA plate cultures using the Maxwell 16 Cell DNA Purification kit (Promega, US). Concentration of extracted DNA was determined using the fluorescent based assays on the Thermo Fisher® Scientific Qubit® fluorometer. Libraries of genome fragments were prepared for sequencing using the Nextera XT v2 set A kit (Illumina, CA, US) as per manufacturer’s instruction. Sequencing was performed on an Illumina NextSeq 500 platform with 150-bp paired-end reading.

The sequenced raw reads were trimmed with Trimmomatic [36] and reads shorter than 20 bp were discarded. The resulting reads were mapped using BWA-MEM to the complete genome of *Escherichia coli* ATCC 8739 and also the two colistin susceptible strains of this study (CREC18, 19) (for identification of altered loci involved in colistin resistance in CREC17). Variant calling process and filtering for high-quality variants were performed as described previously [37].

### Nanopore long-read DNA sequencing and analysis

In order to recover large-size DNA, genomic DNA of CREC strains were purified from liquid culture using the Genomic DNA Clean & Concentrator kit (Zymo Research, Irvine, CA, US), according to the standard protocol (https://files.zymoresearch.com/protocols/). In the next step, rapid barcoding sequencing kit (SQK-RBK004 version 6, Oxford Nanopore) was used according to the manufacturer’s protocol for genomic DNA. The sequencing was carried out on a portable MinION device using a flow cell R9.4.1(Oxford Nanopore). Local basecalling was performed using the Guppy (version 3.1.5) (Oxford Nanopore) with the option enabled to trim the sequencing adapters. NanoFilt program (version 2.0.0) was used to filter out the reads with Phred-score < 7 and a length < 1000 and the statistics of the reads and quality scores were extracted with NanoStat (version 0.8.0). Finally, NanoLyse software (version 0.5.0) was used to remove the control DNA [38].

### Hybrid whole-genome assembly and annotation

In order to reconstruct a high-quality assembled genome and plasmids based on the Illumina short reads and ONT long reads, a hybrid genome assembly was exploited through the following steps. Nanopore long reads were corrected using the Ratatosk de novo error correction tool [39] using Illumina paired-end short reads. In the next step the corrected short and long reads were assembled using Unicycler (version 0.4.8), a widely-used algorithm based on short-long reads hybrid approach [40]. The assemblies were initially visualized by Bandage (version 0.4.8) [41], a program for visualising de novo assembly graphs. For each isolate the complete and closed plasmids were exported for annotation using prokka [42] and the General Feature Format (GFF) file of each plasmid were imported to ApE software (A plasmid Editor, version 3.1.2) to draw the plasmid maps.

Plasmid replicon typing was also performed using the PlasmidFinder (Database version November/2021) with recommended parameters (https://cge.cbs.dtu.dk/services/PlasmidFinder/). The pMLST type of the plasmids were assigned using pMLST 2.0 (https://cge.food.dtu.dk/services/pMLST/). Moreover, MobileElementFinder (https://cge.food.dtu.dk/services/MobileElementFinder/, Software version: v1.0.3 (2020-10-09), Database version: v1.0.2 (2020-06-09)) was used to identify the mobile genetic elements and their relation to antimicrobial resistance genes and virulence factors. In parallel to our variant calling using only the short reads, WGS-based antimicrobial susceptibility testing was performed on hybrid assemblies using the ResFinder 4.1 with default parameters (https://cge.food.dtu.dk/services/ResFinder/) to detect chromosomal point mutations and acquired antimicrobial resistance.

The sequence reads of all strains were submitted to the NCBI sequence read archive with Project number PRJNA809646. The accession numbers for the publicly available illumina short reads and ONT long reads are as follows respectively: CREC-17(SRR21721852), CREC-18(SRR21721851 and SRR21721848), CREC-19 (SRR21721850 and SRR21721847) and CREC-20 (SRR21721849 and SRR21721846).

## Supplementary Information

**Additional file 1: Supplementary Figure 1.** mCIM results for four carbapenem resistance *E .coli* isolates (CREC-17 to CREC-20) (positive results) and one carbapenem susceptible *E. coli* (CSEC-85) (negative result).

**Additional file 2: Supplementary Table1.** General characteristics of assembled genomes obtained from CREC-18, CREC-19 and CREC-20.

**Additional file 3: Supplementary Figure 2.** Circular visualization of the comparative genome analysis of CREC-18, CREC-19, CREC-20 with *E. coli* ST648 (Genbank Accessions CP048107, as reference genome), *E. coli* ST167 (Genbank Accessions CANDYB000000000) and *E. coli* ST361 (Genbank Accessions CP103704).

**Additional file 4: Supplementary Table 2.** Comparative genome analysis of CREC-18, CREC-19, CREC-20 with *E. coli* ST648 (Genbank Accessions CP048107, as reference genome), *E. coli* ST167, *E. coli* ST167 (Genbank Accessions CANDYB000000000) and *E. coli* ST361 (Genbank Accessions CP103704).

**Additional file 5: Supplementary Figure 3.** Alignment results of two NDM-5 carrying plasmids pCREC-18 and pCREC-20_2.

## Data Availability

The sequence reads of all strains can be accessed in GenBank under accession numbers SRR21721846 to SRR21721852.
