# Supplementary figures and images for: Whole-genome sequence analysis of clinically isolated carbapenem resistant Escherichia coli from Iran
Source: BMC Microbiol. 2023 Feb 27;23:49. doi: 10.1186/s12866-023-02796-y (PMC9969672; doi:10.1186/s12866-023-02796-y)

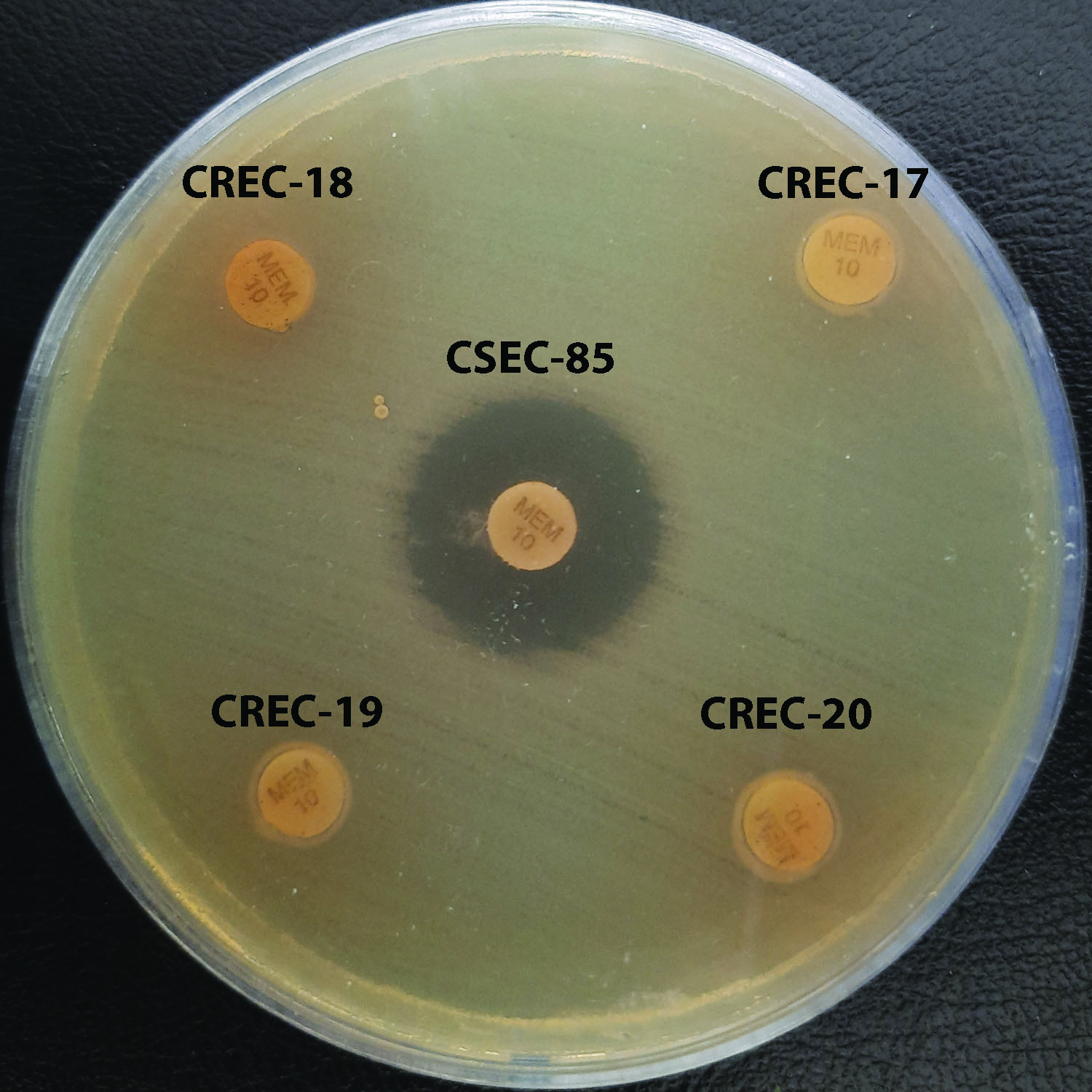

Supplement: Supplementary file 1 — Additional file 1: Supplementary Figure 1. mCIM results for four carbapenem resistance E .coli isolates (CREC-17 to CREC-20) (positive results) and one carbapenem susceptible E. coli (CSEC-85) (negative result). [file 12866_2023_2796_MOESM1_ESM.jpg]

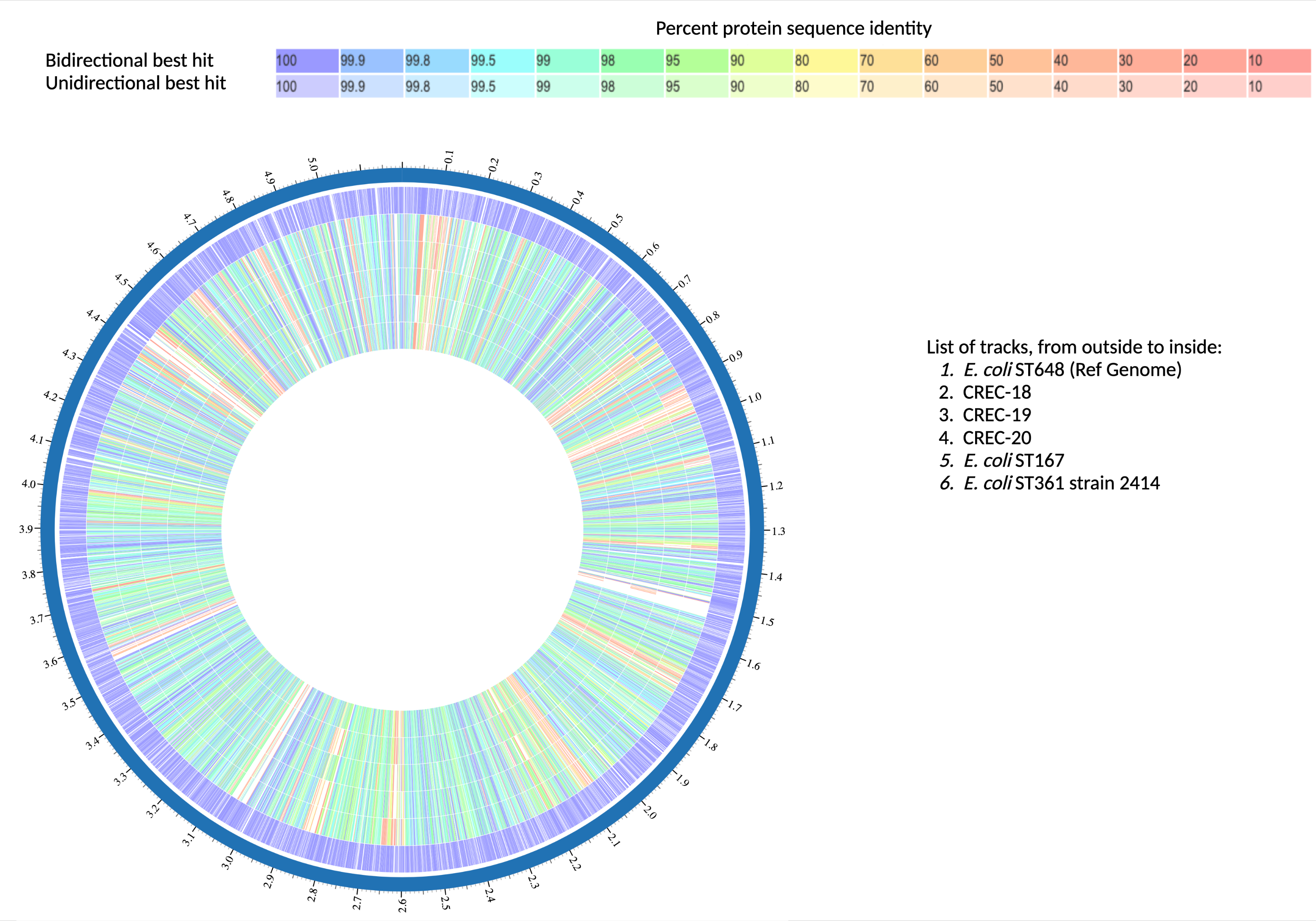

Supplement: Supplementary file 3 — Additional file 3: Supplementary Figure 2. Circular visualization of the comparative genome analysis of CREC-18, CREC-19, CREC-20 with E. coli ST648 (Genbank Accessions CP048107, as reference genome), E. coli ST167 (Genbank Accessions CANDYB000000000) and E. coli ST361 (Genbank Accessions CP103704). [file 12866_2023_2796_MOESM3_ESM.jpg]

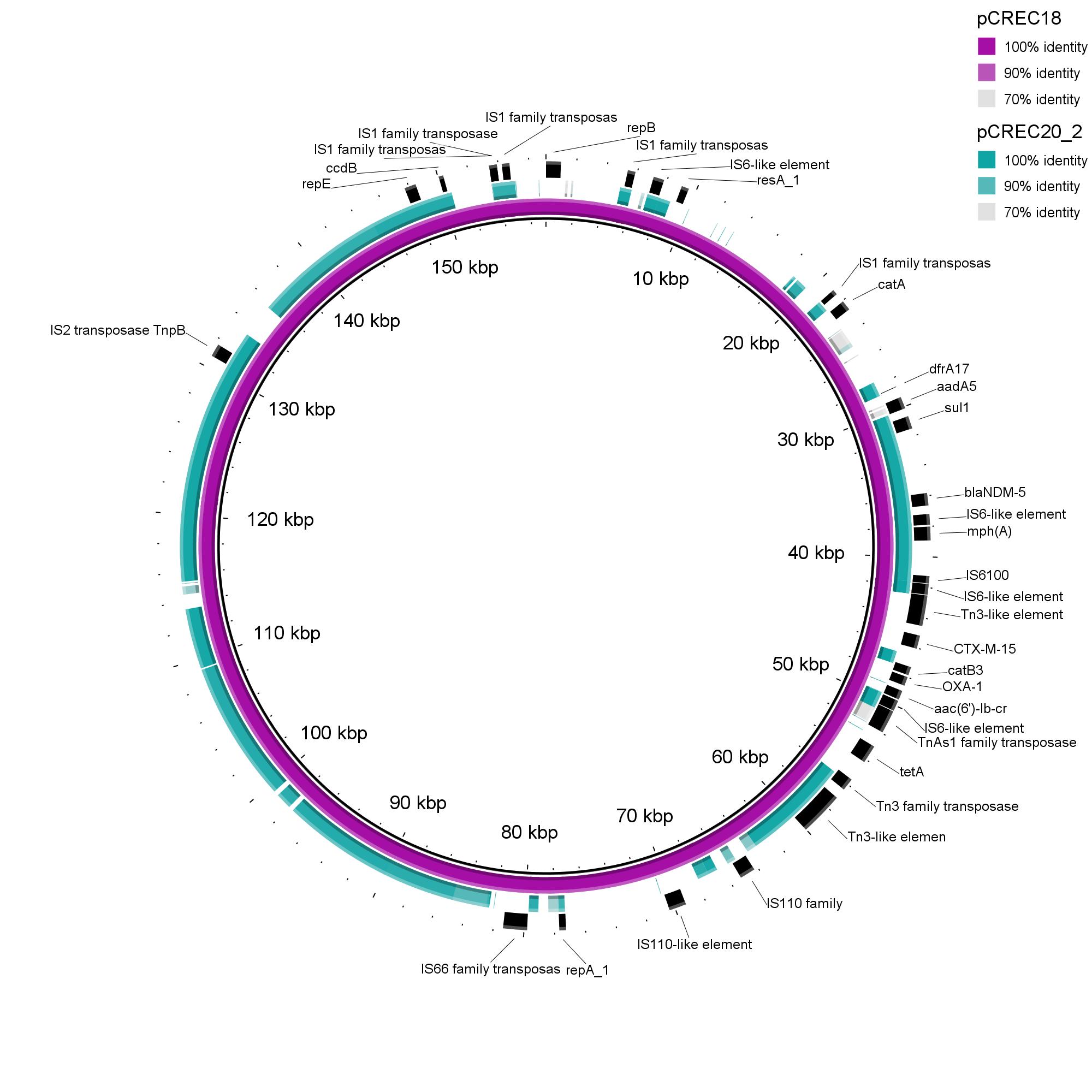

Supplement: Supplementary file 5 — Additional file 5: Supplementary Figure 3. Alignment results of two NDM-5 carrying plasmids pCREC-18 and pCREC-20_2. [file 12866_2023_2796_MOESM5_ESM.jpg]
